# Analysis of the size reduction of AgNPs loaded hydrogel and its effect on the anti‐bacterial activity

**DOI:** 10.1049/nbt2.12037

**Published:** 2021-03-22

**Authors:** Michelle Dsouza, Sakthi Swarrup Jayabalan

**Affiliations:** ^1^ Centre for Nanotechnology Research Vellore Institute of Technology Vellore India

## Abstract

This article analyses the effect of the size reduced Silver (Ag) loaded hydrogel by (a) lyophilisation (S1) (b) ball milling (S2) techniques and its effect on anti‐bacterial activity. The g loaded hydrogel, S1 and S2 shows an increase in swelling with an increase in pH. The swelling is more for Ag loaded hydrogel in low pH. For pH above 7, the swelling ratio of Ag loaded hydrogel and S1 are almost the same while S2 shows very less swelling. The anti‐bacterial studies reveal that S1 and Ag loaded hydrogel reacted well in S. aureus (Staphylococcus aureus) but no zone formation was seen in S2 .whereas no zone was formed in S1 and S2 for E‐coli (Escherichia coli). As the next step, the anti‐bacterial activity of Ag loaded hydrogel with the addition of curcumin (CS1―size reduced by lyophilisation, CS2―size reduced by ball milling) and turmeric (TS1―size reduced by lyophilisation, TS2―size reduced by ball milling) were investigated. In case of E.coli, a zonal formation of 1.2 cm for TS1 and 1.1 cm for TS2 and 1 cm for CS1 and 0.2 cm for CS2 was observed. For S.aureus, 1.1  and 1 cm were seen for TS1 and CS1. TS2 and CS2 did not show any zone formation. These studies clearly show that size reduction by lyophilisation (S1, TS1 and CS1) is more efficient in all the cases when compared to the ball milling technique (S2, TS2 and CS2). Comparing TS1 with S1 and CS1, TS1 has highly efficient/effective anti‐bacterial properties than S1 and CS1. Therefore, lyophilised hydrogel incorporating turmeric and silver (TS1) is an excellent choice compared to using curcumin for wound dressing applications.

## 1 INTRODUCTION

Over the past decade, a variety of new therapies are available for patients with acute and chronic wounds [1], such as sensor embedded dressings and metal oxide dressings; sensor embedded dressing is short lived and costly, whereas the metal oxide used in high amount could be harmful for the wound [2, 3]. An alternative to overcome these disadvantages is to use hydrogel for wound dressings. Hydrogels are hydrophilic in nature. These hydrogels can be applied over the wound and removed very softly [4]. They have a cooling specialisation factor which decreases the temperature of the wound. Therefore, hydrogels are well‐suited for wound dressing applications [5]. They are fundamentals in tissue engineering as they promote tissue regeneration. Other advantages of hydrogels include their biocompatible they are biocompatible, biodegradable and cost‐effective [6]. Due to their properties like high biocompatibility and biodegradability, hydrogels have been a subject of attraction.

During the past 20 years, several technologies have been put into use, asnanoparticles can be stabilised by use of micro and nano hydrogels as well as polymers [7]. Hydrogel nanocomposites have the capacity to absorb large amount of water in cross‐linked networks, due to their hydrophilic nature. Therefore, they form highly cross‐linked hydrophilic networks with metal nanoparticles like silver, gold, zinc, copper and titanium. These nanoparticles increase the antibacterial efficiency of the hydrogel [8, 9]. This nature of hydrogel makes it highly effective in biomedical applications [10].

Hydrogel nanocomposites can be classified into two categories, inorganic nanoparticles containing hydrogels and antibacterial agents containing hydrogels. The inorganic nanoparticles containing hydrogels include metal oxides formed from silver (AgO) and copper (CuO). Apart from these metals, zinc, nickel and titanium have also been used with hydrogels in their oxide forms owing to their efficient anti‐bacterial properties [11]. Similarly, gold (Au) plays a significant role in biomedical applications due to their special qualities of sensing bacterial activities. Other metallic compounds used with hydrogels are cobalt combined with zeolites. These compounds have shown excellent anti‐bacterial properties on the E.coli bacteria [11]. However, recent studies have also stated the negative effects of the metal oxides formed. Formation of these metal oxides requires the use of highly toxic chemical processes that are harmful for the environment and in turn for humans [12]. Copper and zinc oxides have been reported as the most toxic metal oxides, followed by titanium which is less toxic compared with the others. The use of copper and zinc in anti‐microbial agents kills the bacterial cell, but in turn is harmful for humans as it causes mitochondrial damage and oxidative stress. Seabra AB and Duran N in their study have ranked these metal oxides on the basis of their toxicity levels such as ZnO > CuO > TiO_2_ [12].

Therefore, in comparison to the above metals, silver is considered to be the most effective metal that can be used for anti‐bacterial activities, due to its antimicrobial and anti‐inflammatory activities. Silver and silver ions are widely known for their bacterial and fungicidal activities. Silver has been exploited for its medicinal properties for centuries [13, 14]. It is chosen for its anti‐bacterial therapeutic values on account of its acknowledged low toxicity and as an anti‐microbial agent that controls S. aureus and E.coli bacteria. Studies lately have shown that silver nanoparticles usually within the range of 1 to 10 nm exhibit strong anti‐bacterial properties [14]. They have the ability to reach numerous sites at a time [15]. However, it causes toxicity or biological hazards if used in large quantities [16, 17]. Similar to silver, turmeric is a naturally occurring compound used for its anti‐bacterial purposes. The anti‐microbial properties of turmeric are due to the presence of curcuminoids, (which give them the yellow colour), turmerol, turmeric oils and veleric acid. Similarly, curcumin, a compound of turmeric is extracted from the rhizome of Curcuma longa by various solvent extraction methods using ethanol [18]. Curcumin is the main active ingredient of turmeric, which possess a wide range of biological activities which may include wound healing, anti‐bacterial, anti‐oxidant, anti‐inflammatory and anti‐cancer, anti‐fungal and anti‐viral properties [19].

In the present work, a new approach to improve the anti‐bacterial properties is attempted by reducing the size of the hydrogel loaded with silver, turmeric and curcumin. The effects of using turmeric (which contains few other ingredients along with curcumin) and only the curcumin extracted from turmeric were investigated. Comparative studies on the effect of size reduction on the swelling behaviour at different pH and on the anti‐bacterial activity in Escherichia‐coli (E‐coli), and Staphylococcus aureus (S. aureus) were carried out.

## 2 MATERIALS USED FOR THE STUDIES

The hydrogel , Polyacrylamide (PAm) used for the present study, was extracted from disposable diapers. The extracted hydrogel is present at the core of the diapers and its main function is to soak up the liquid and keep moisture away. Silver nitrate (AgNO_3_) of molecular weight 169.87 mg, sodium borohydride (NaBH_4_) of molecular weight 37.8 mg, turmeric (naturally obtained), curcumin (a compound of turmeric that is. extracted from the rhizome of Curcuma longa), HCL, NaOH and phosphate buffer solutions of pH 7.4 were purchased from Sigma Aldrich.

## 3 FABRICATION OF ANTI‐BACTERIAL SILVER (Ag) LOADED HYDROGEL AND SIZE REDUCED AgNPs LOADED HYDROGEL

### 3.1 Analysis on the effect of pH on the swelling behaviour of extracted hydrogel

As the wound pH is a monitoring factor for wound healing, the effect of pH on the swelling behaviour of hydrogel was studied. About 1 μL of diluted HCL and diluted NaOH solution were prepared and mixed with 38 ml water in two separate beakers. These solutions were brought to standard pH values of a wound (4‐10) by increasing or decreasing the amount of acid or base, which was measured by a pH meter. One millilitrel of each solution of standard pH (4‐10) values was added to 0.1 g of Ag loaded hydrogel and the difference in their weight was measured by the formula [10]:

$$\text{Swelling}\;\text{ratio}=\frac{\text{Weight}\;\text{of}\;\text{swelled}\;\text{gel}\left(g\right)‐\text{Weight}\;\text{of}\;\text{initial}\;\text{gel}\left(g\right)}{\text{Weight}\;\text{of}\;\text{initial}\;\text{gel}\left(g\right)}$$

### 3.2 Loading of silver (Ag) into the hydrogel

**FIGURE 1:**
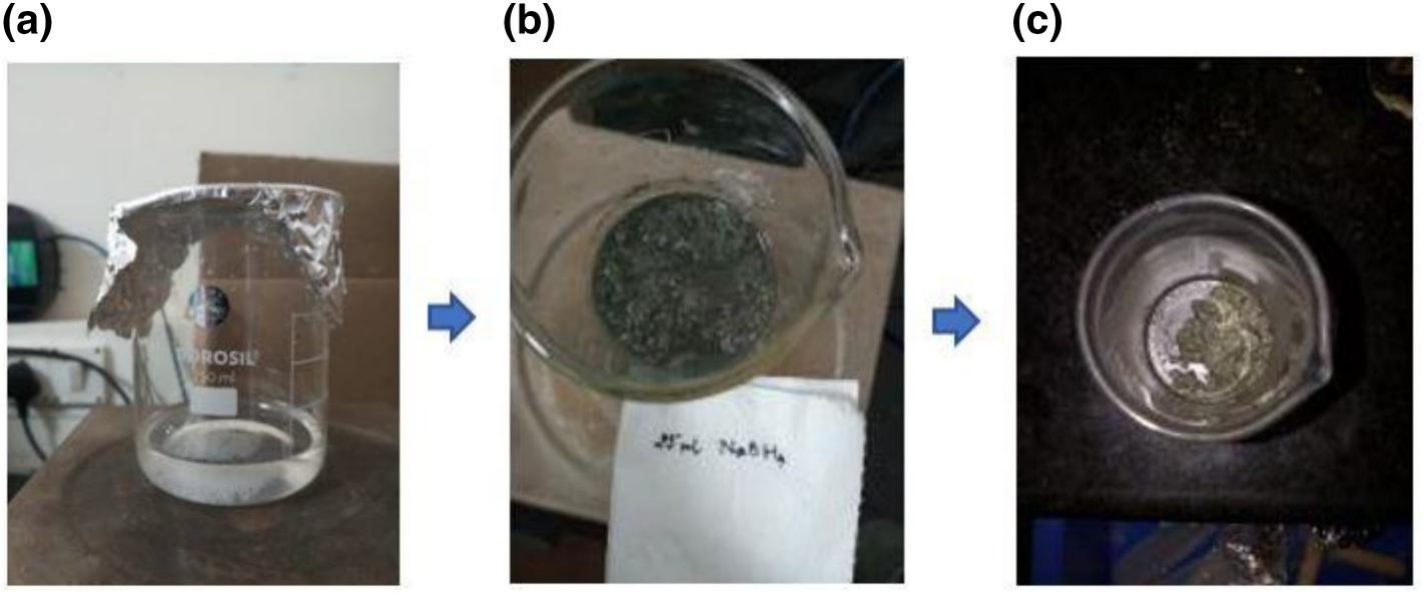
Images showing the incorporation of silver nanoparticles into the hydrogel

The procedure for loading Ag into the hydrogel was adapted from Panáček et al. [15]. As the first step, 50 mg of hydrogel was allowed to swell in 30 ml of water. A 30 ml silver nitrate solution was prepared using 5 mM of silver nitrate. The swollen hydrogel was then added to the silver nitrate solution. The solution was left undisturbed for 24 h for the silver ion to get loaded into the hydrogel. A 25 ml solution was prepared from 5 mM sodium borohydride and added to the above prepared silver loaded hydrogel. After 24 h, the silver loaded hydrogel changes its colour due to its reaction with sodium borohydride (Figure 1). The silver loaded hydrogel obtained after this step was used for the further analysis in the paper.

### 3.3 Size reduction of Ag loaded hydrogel using the lyophilisation process (S1) and ball milling (S2)

The size reduction of Ag loaded hydrogel was carried out using a Lyodel freeze dryer (Delvac pumps Pvt. Ltd) by freezing it at a temperature (−50°C) and pressure 0.2 bar. Further sublimation was done to remove the water and a dry gel was obtained. Another technique for size reduction was carried out by the planetary ball milling machine (VBCC, Chennai) by placing the hydrogel in a hollow cylindrical rotating shell with 20 steel balls. The sample was rotated at a speed of 250 rpm for 8 hours. The size reduction of the sample took place by the combined mechanism of rotation of the shell and the movement of the balls.

### 3.4 Studies on the swelling ratio of Ag loaded and size reduced Ag loaded hydrogel

**FIGURE 2:**
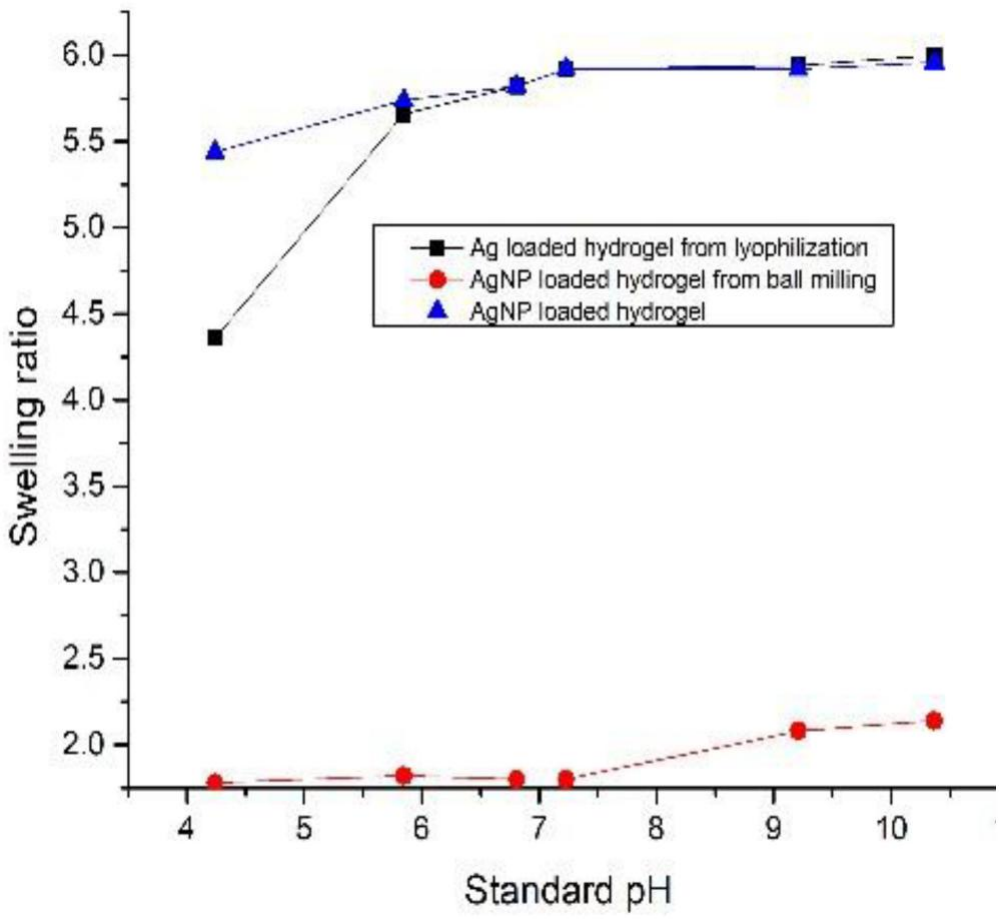
Comparison of and Ag loaded hydrogel, S1 (lyophilised) and S2 (ball milled) at standard pH values

The swelling ratio of Ag loaded hydrogel and S1 increased initially for pH values four to 6. Initially, the swelling ratio of Ag loaded hydrogel was 5.4 for pH four and increased to 5.9 at pH whereas for S1 swelling ratio for pH four was 4.3 and pH six was 5.4. However, at pH 10, the swelling ratio of Ag loaded hydrogel reduced to 5.6, whereas the swelling ratio of S1 increased to 6. This shows that swelling ratio S1 at a higher pH (10) was more when compared with swelling ratio of Ag loaded hydrogel (Figure 2), S2 which was very low at standard pH values.

### 3.5 Characterisation of Ag loaded and size reduced Ag loaded hydrogel

#### 3.5.1 UV spectroscopy analysis

**FIGURE 3:**
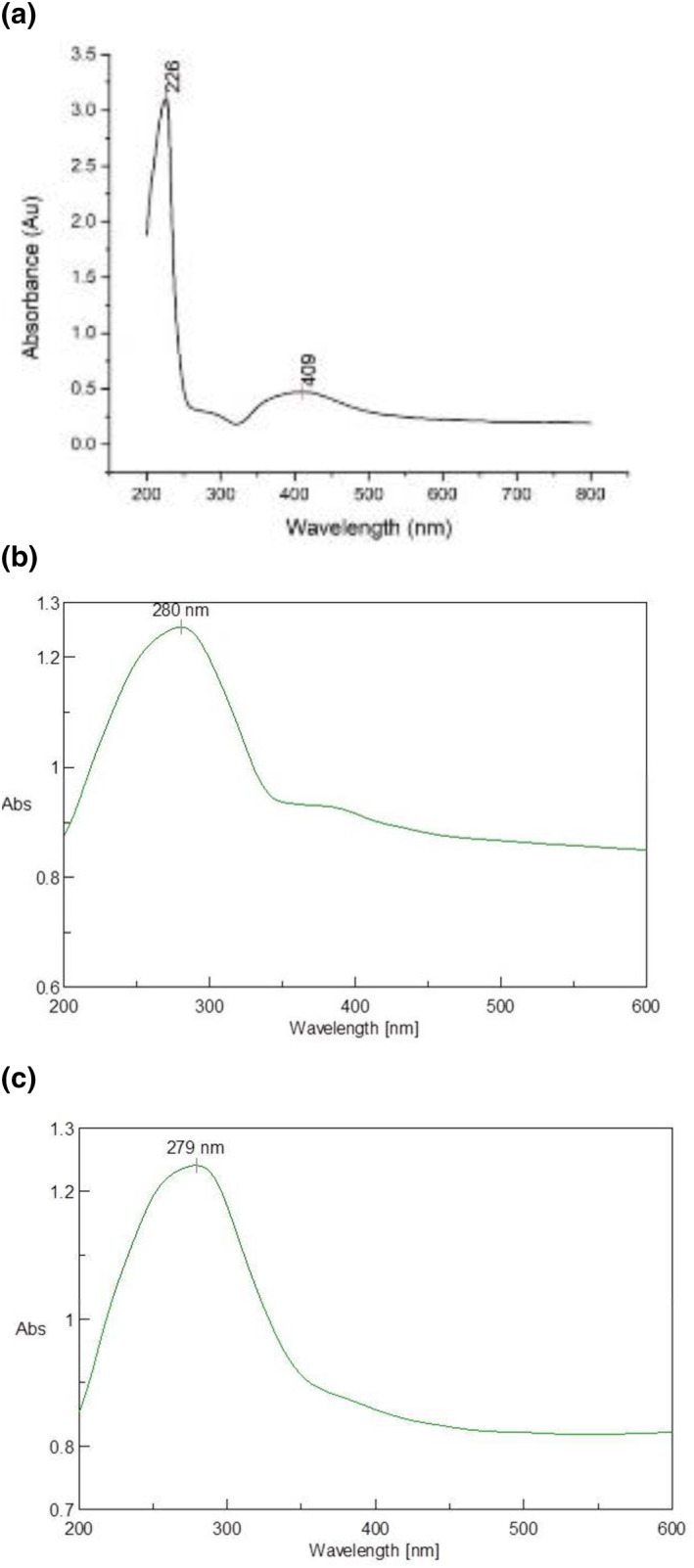
UV spectroscopy graph of (a) AgNPs loaded hydrogel (b) S1 and (c) S2

Figure 3a,b,c represents the UV‐vis/Vis spectroscopy of the Ag nanoparticle loaded hydrogel, S1 and S2. For S1 and S2, the peaks were obtained at 280  and 279 nm. The shift in the Ag peaks could be attributed to the aggregation of the silver nanoparticles and can be rationalised by decreasing the swelling of silver ions before reduction. This states that loading conditions of silver ions before their reduction plays an essential role in the morphology of the silver nanoparticles [8, 9].

#### 3.5.2 X‐ray diffraction studies

**FIGURE 4:**
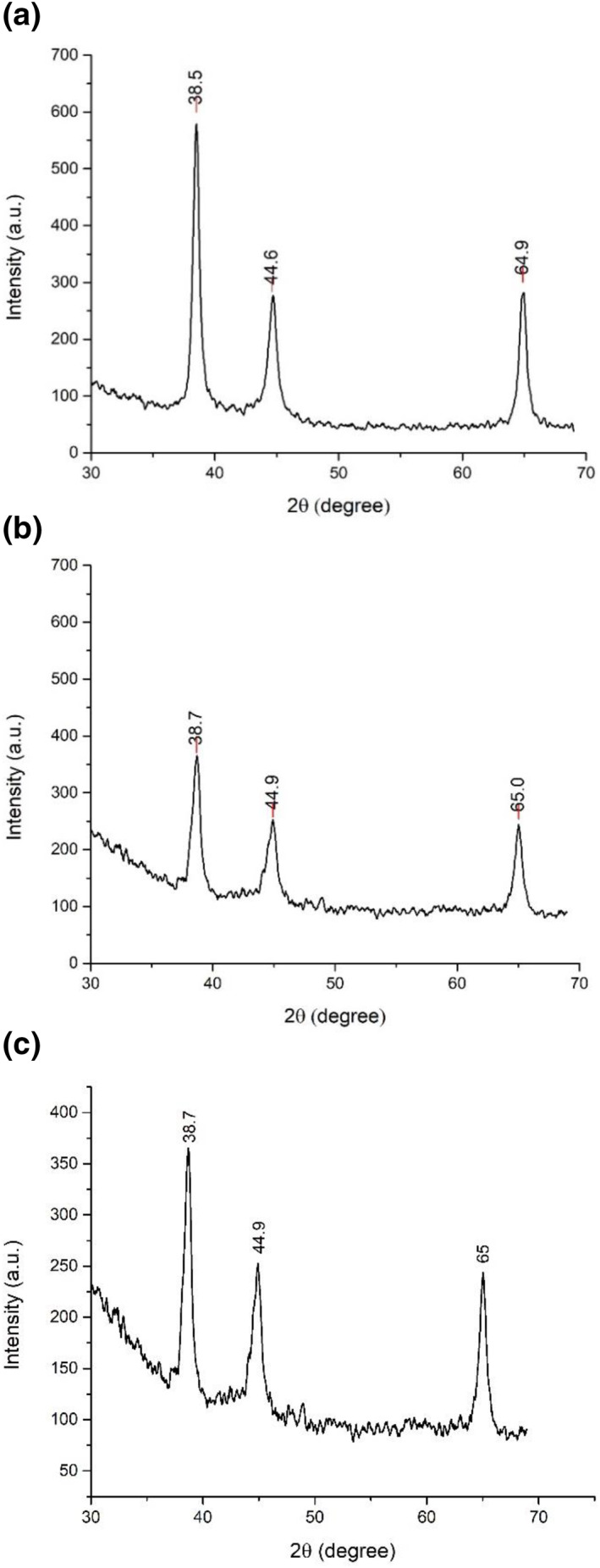
XRD peaks of (a) Ag loaded hydrogel (b) S1 (c) S2

Figure 4a,b illustrates the X‐ray diffraction (XRD) pattern of silver nanoparticles loaded hydrogels, S1 and S2, respectively. The observed peaks of diffraction for both the silver composites were found at 2*θ* = 38.13, 44.12, 64.38 and 77.35, assigned to planes (111), (200), (220) and (311) of face centred cubic unit cell. Results stated that broad peaks in XRD generally refer to smaller particle size whereas the obtained sharp peaks represent the highly crystalline silver nanoparticles that are formed in the hydrogel [10].

**FIGURE 5:**
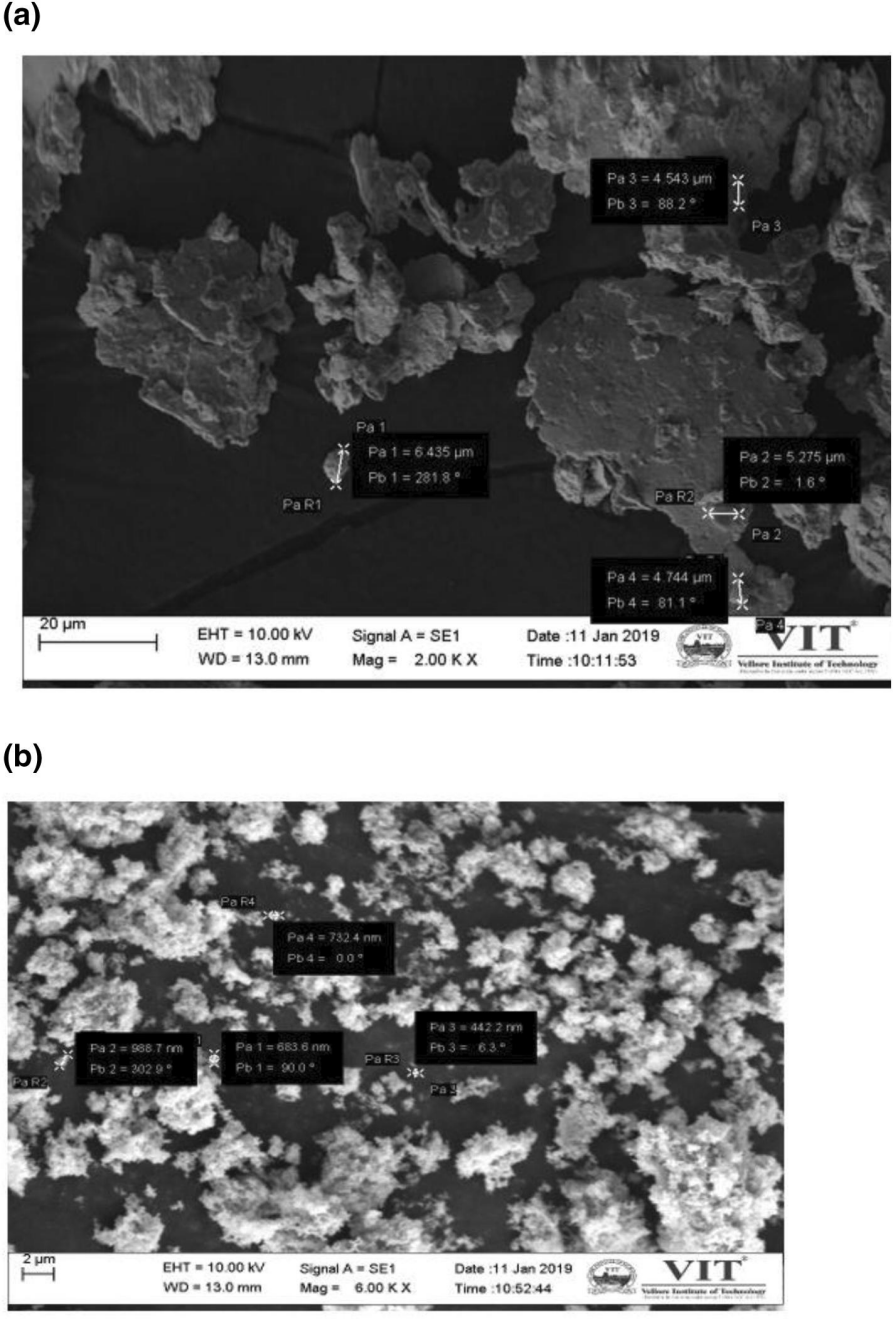
Particle size of (a) S2 is (4.5–6.5) μm and (b) S1 is (0.4–0.9) μm

**FIGURE 6:**
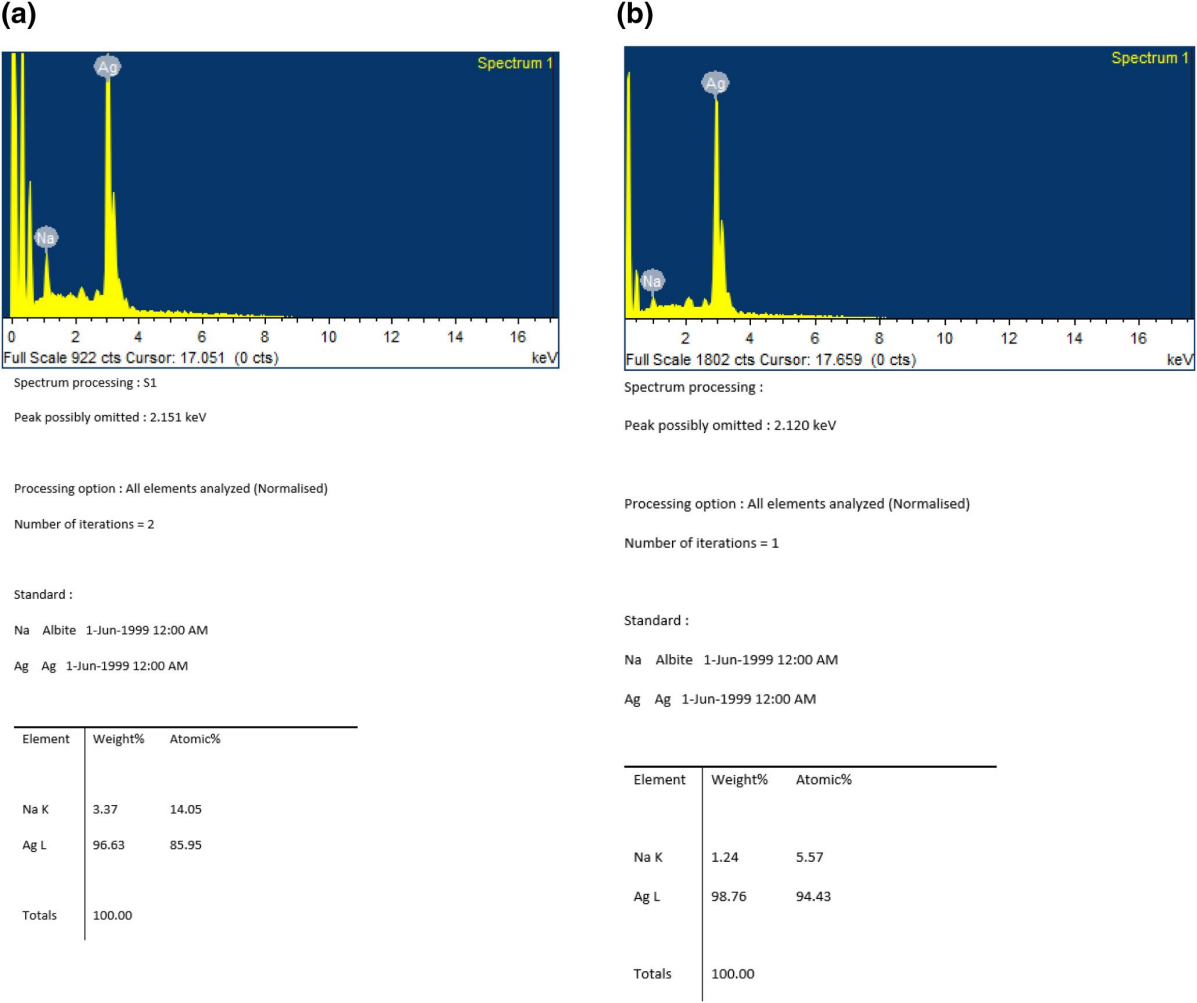
Energy dispersive X‐ray analysis of (a) S2 and (b) S1

SEM analysis of S1 and S2: SEM analysis of size reduced AgNP loaded gels was carried out to find the particle size and composition of silver particles present in the gel (Figures 5 and 6).

### 3.6 Anti‐bacterial studies of AgNPs loaded hydrogel and size reduced AgNPs loaded hydrogel (S1 and S2)

Hydrogel‐based wound dressings against Escherichia‐coli (E‐ coli), and Staphylococcus aureus (S. aureus) bacteria illustrated the anti‐bacterial effect of non‐reduced silver nanoparticles loaded hydrogel on both the bacteria [20, 21]. Studies indicate that the non‐reduced silver nanoparticles of the loaded hydrogel exhibit a great reduction in the growth of both E‐coli as well as S. aureus bacteria. The silver nanoparticles released in the hydrogel combine with the sulphur present in the wound bacteria, which in turn kills the bacteria [22].

For anti‐bacterial studies, Muller Hinton Agar (MHA) plates were prepared a day before the test. About 24 h later the MHA plates were swabbed with S. aureus (gram+) and E‐coli (gram‐) bacteria and tiny wells were bored in plates for the test sample and negative control (water). The test sample and negative control were added in equal amount (100 μL) in the holes. The positive control (antibiotic) was placed on the plates and it was left for 24 h for zonal formation [6, 7].

**FIGURE 7:**
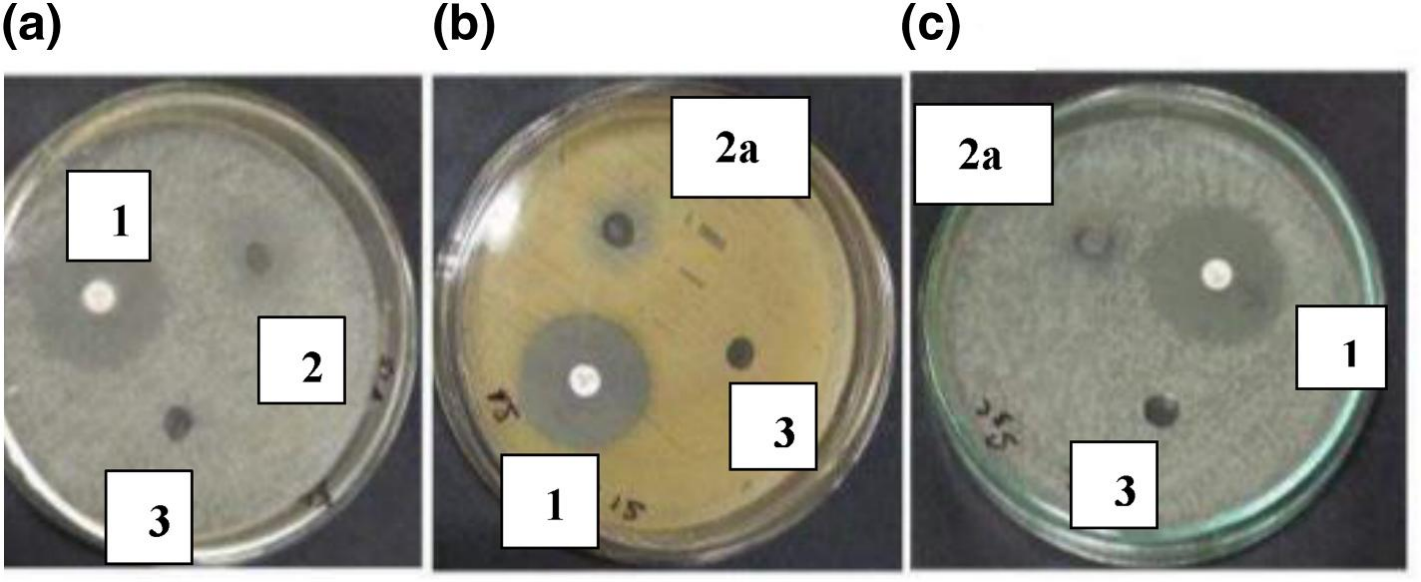
AgNP loaded hydrogel on (a) E‐coli, (b) Staphylococcus aureus bacteria and (c) S1 on Staphylococcus aureus.

**FIGURE 8:**
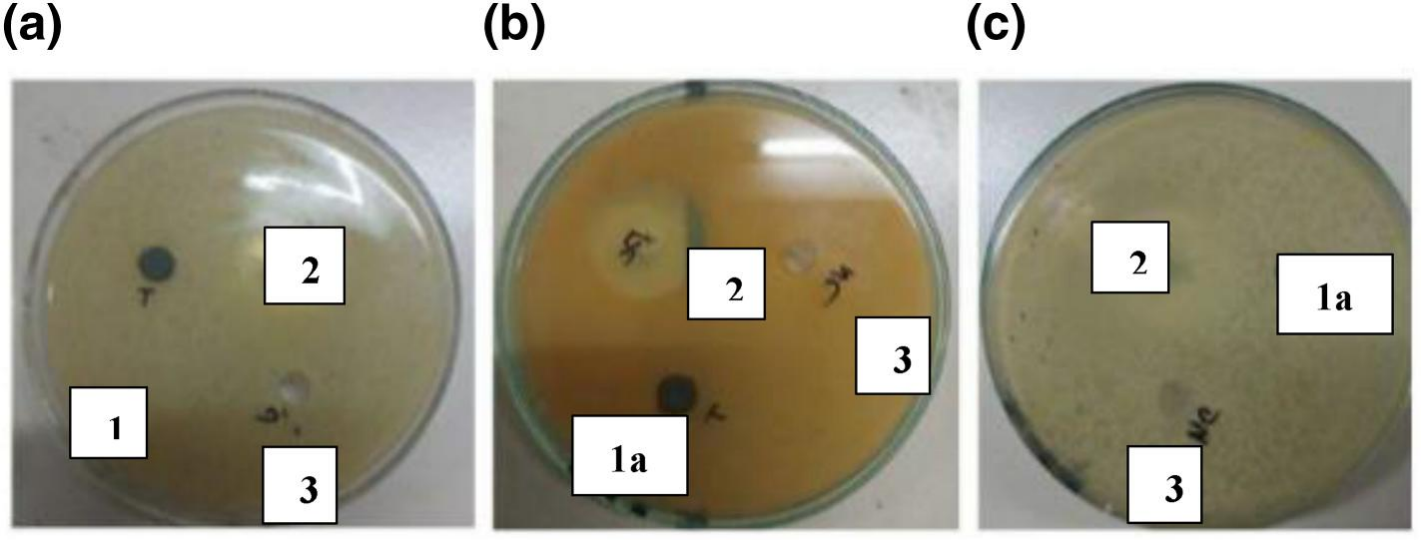
Anti‐bacterial activity of (a) S1 on E‐coli, (b) S2 on S. aureus and of (c) S2 on E‐coli

The results clearly showed restrictions in the formation of bacterial colonies in the non‐reduced silver nanoparticle loaded hydrogels, whereas the size reduced silver nanoparticle loaded hydrogels showed little to no restriction. The results showed similar trends to the already published literature [23]. S1 has more capability of retarding bacterial growth compared with S2. This is due to the ion exchange mechanism that takes place between the silver nanoparticles present in silver nitrate [24]. The ‐COOH groups present in the hydrogel are the main source for formation of silver nanoparticles. The reduction of silver loaded gel by sodium borohydride (NaBH4) also has an effect on the bacterial action [25]. Table 1 revealed that silver was a more active composite in silver nanoparticles loaded hydrogel compared to lyophilised and ball mill reduced AgNP loaded hydrogel. Figures 7 and 8, show the effect of non‐reduced and reduced silver nanoparticles loaded hydrogels, respectively, towards the bacteria.

**TABLE 1:** Zone formation for E.coli on silver nanoparticles loaded hydrogel, S1 and S2

Name of the sample	Diameter of zonal (cm)‐ (E‐coli)			Diameter of zonal (cm) – S.Aureus		
	Without size reduction	S1	S2	Without size reduction	S1	TS2
Test sample	0.5	No zone	No zone	0.3	0.25	No zone
Positive control (antibiotic)	1	1	1	1	1	1
Negative control(water)	No zone	No zone	No zone	No zone	No zone	No zone

The samples used in Figure 7 for the anti‐bacterial analysis of silver nanoparticle loaded hydrogel and S1 are: (1) positive control or antibiotic, (2) Ag nanoparticle loaded hydrogel, (2a) S1 and 3) negative control or water.

The samples used in Figure 8 for the anti‐bacterial analysis of S1 and S2 are: (1) S1, (1a) S2, (2) positive control or antibiotic and (3) negative control or water.

Zone formation on MHA plates for silver nanoparticles loaded hydrogel, and size reduced silver nanoparticles loaded hydrogel (S1 and S2) with water as the negative control on Escherichia‐coli (E‐coli)

Table 1 is a tabular representation of the zone formation on MHA plates on silver nanoparticles’ loaded hydrogel, silver nanoparticle loaded hydrogel obtained as S1 and S2 with water as the negative control on Escherichia‐coli (E‐coli) and Staphylococcus aureus bacteria.

## 4 IMPREGNATION OF ANTI‐BACTERIAL AGENTS (a) TURMERIC (b) CURCUMIN INTO THE SIZE REDUCED AG LOADED HYDROGEL

TS1 and TS2 represent the size reduced Ag loaded with turmeric hydrogel using lyophilisation and ball milling. However, CS1 and CS2 indicate the size reduced Ag loaded with curcumin hydrogel using lyophilisation and ball milling.

a) Turmeric

About 50 mg of the hydrogel was taken in 20 ml of water and allowed to swell. A solution of silver nitrate was further prepared by adding 5 mM silver nitrate to 50 ml of water. Another solution was prepared by adding 5 mg turmeric (a naturally obtained compound) to 20 ml solution of acetone and water in the ratio 4: 6. The swollen hydrogel was taken out and added to 50 ml of AgNO_3_ solution. Later, the 20 ml turmeric solution was added to the AgNO_3_ solution and left for 24 h [23]. The hydrogel is collected after 24 h for further analysis [23].

b) Curcumin

For loading the curcumin, 50 mg of dry hydrogel was allowed to swell in 20 ml of curcumin solution. Five milligram of curcumin (a compound of turmeric i.e. extracted from the rhizome of curcuma longa) was added to solution of acetone: water in the 4: 6 (8 ml of acetone and 12 ml of water) ratio and left for 24 h at 25°C temperature [23].

### 4.1 Characterisation studies of (a) turmeric (b) curcumin impregnated size reduced Ag loaded hydrogel (TS1, TS2 and CS1, CS2)

#### 4.1.1 UV‐vis spectroscopy analysis

The absorption peaks of the AgNP loaded hydrogel and curcumin or turmeric AgNP loaded hydrogel lies within the range of 200  to 410 nm. From the graphs it can be observed that the addition of curcumin or turmeric to AgNP loaded hydrogel increases the reduction and stabilisation capabilities of the formed nanoparticles which are due to the presence of O‐H (hydrophilic) groups. Spectroscopy surface plasmon effect determines the absorbed silver nanoparticles. A shift takes place in the wavelength of the spectra as curcumin or turmeric is added individually to the AgNP loaded hydrogel. As observed from the Figure 7 with the addition of these components into AgNP loaded hydrogel the UV absorbency value decreases . The reason for this is that the cross‐linking that occurs between silver and curcumin bonds and silver and turmeric bonds. This causes reduction in the free hydrogel networks and reduces the number of silver nanoparticles formed in the hydrogel network.

**FIGURE 9:**
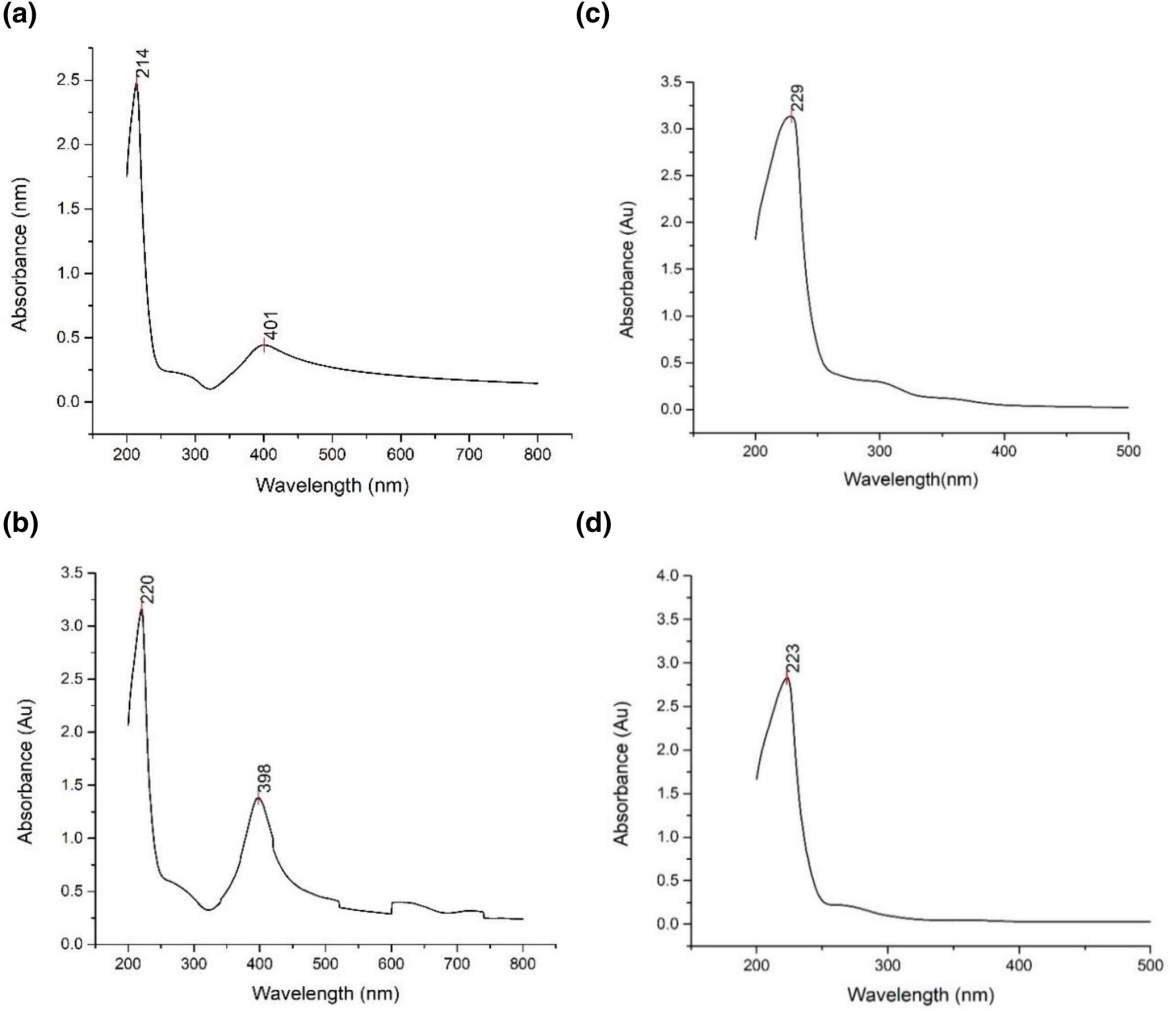
UV‐Vis peaks for (a) TS1, (b) CS1, (c) TS2 and (d) CS2

Figure 9a,b,c,d represents UV‐ vis spectroscopy analysis TS1, CS1, TS2 and CS2 in 7.4 pH phosphate buffer.

#### 4.1.2 Fourier transform infrared spectroscopy analysis

The Fourier transform spectra of the samples were recorded on a Fourier transform infrared spectroscopy (FTIR) spectrophotometer (SDT Q600) using KBr. FTIR analysis is carried out to indicate the interaction between a metal and composites or metal and polymers [21].

**FIGURE 10:**
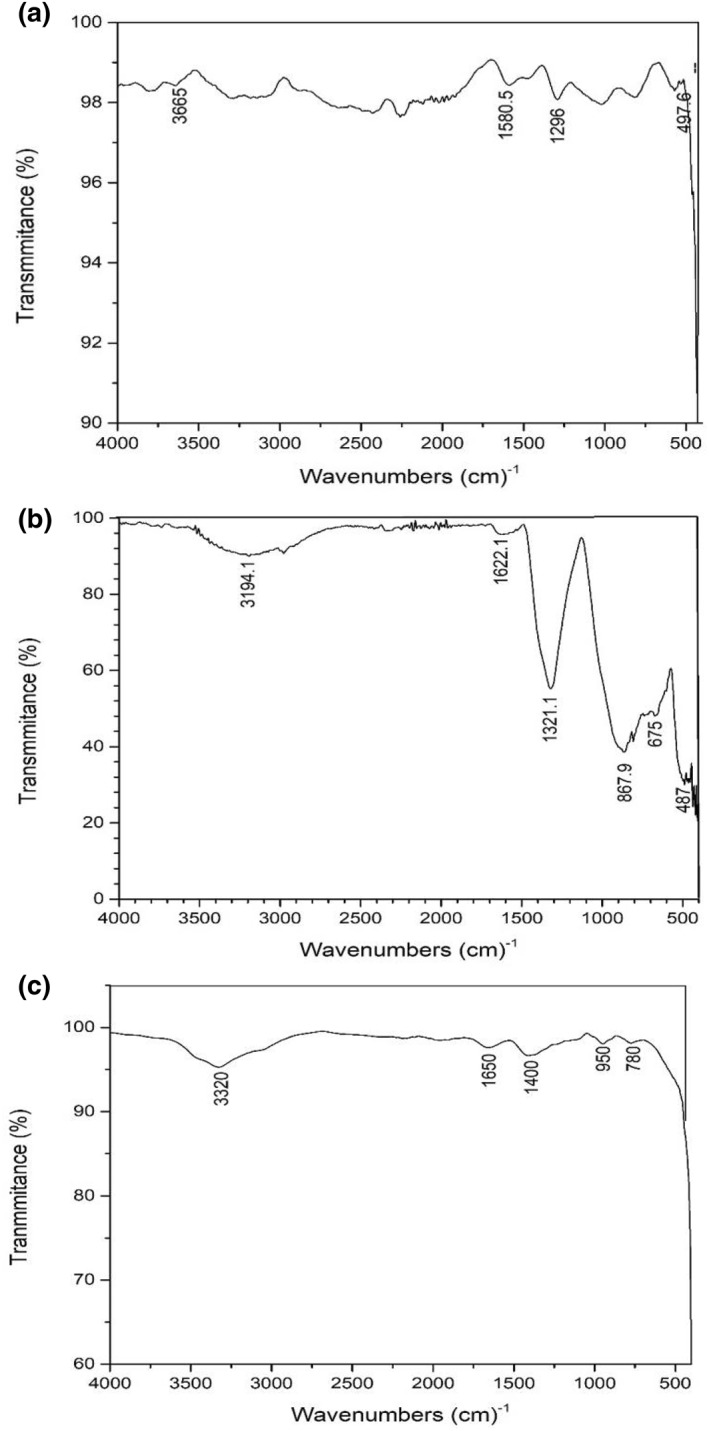
Fourier transform infrared spectroscopy bands for (a) Ag loaded hydrogel (b) TS1

**FIGURE 11:**
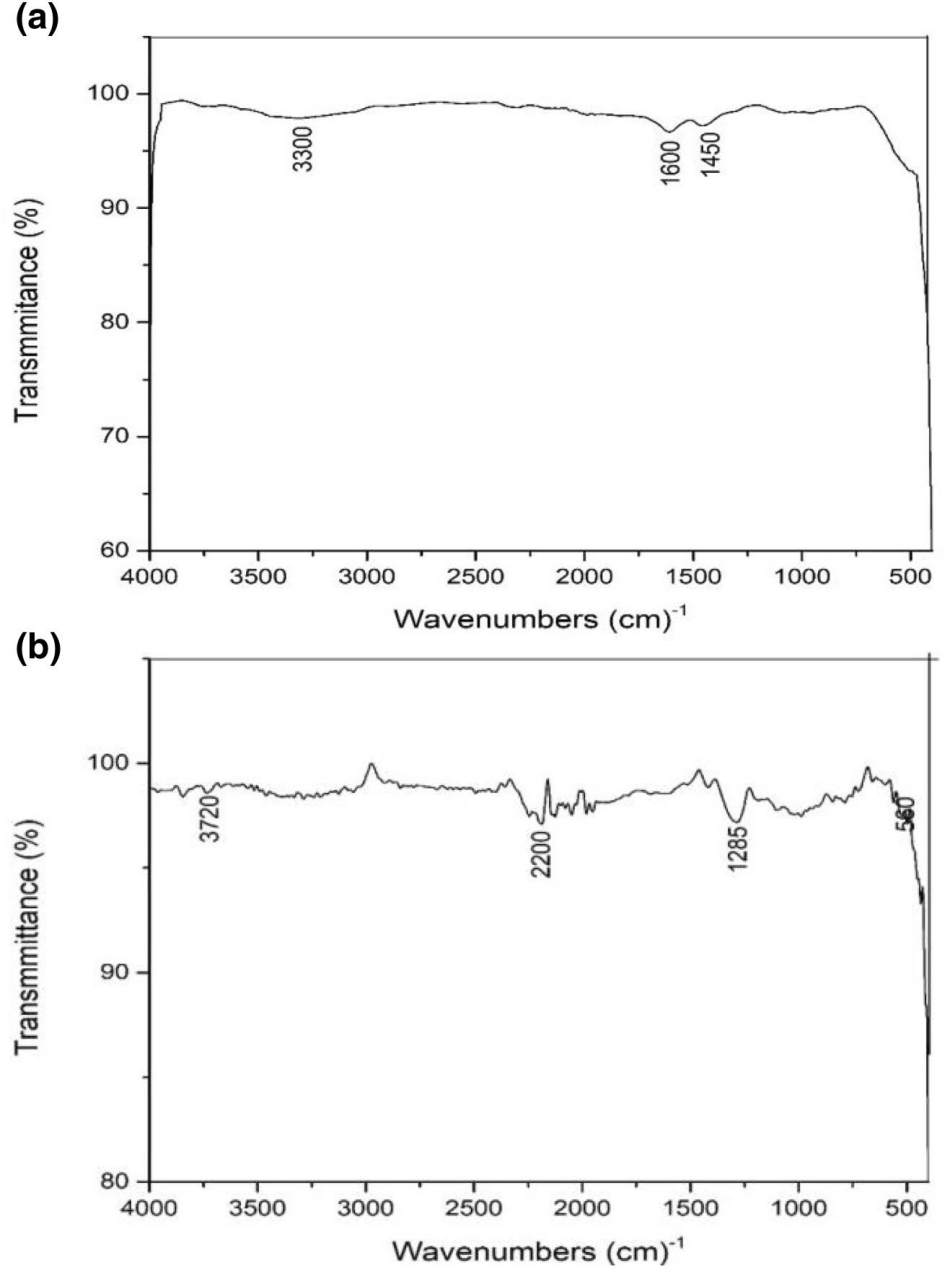
Fourier transform infrared spectroscopy bands for (a) CS1 (b) CS2

In the present study, FTIR analysis was carried out to identify the incorporation of silver nanoparticles or the amount of silver absorbed within the gel network. The size reduced samples (TS1, TS2, CS1 and CS2) are compared with Ag loaded hydrogel to see the shift in bands due to size reduction. The peak characteristics were shown for AgNP loaded hydrogel at band regions 497.6 cm^−1^, 1296 cm^−1^, 1580.5 cm^−1^ and 3665 cm^−1^ thus confirming the presence of silver in the network. Peaks were observed for TS1 at 467.6 cm^−1^, 675.09 cm^−1^, 867.97 cm^−1^, 1321.24 cm^−1^, 1622.13 cm^−1^ and 3194.12 cm^−1^. Peaks were observed for TS2 are 3320 cm^−1^, 1650 cm^−1^, 1400 cm^−1^, 859 cm^−1^ and 780 cm^−1^. Peaks were observed for CS1 are 3300 cm^−1^, 1600 cm^−1^, 1450 cm^−1^. Similarly, the bands for CS2 are 3720 cm^−1^, 2200 cm^−1^, 1285 cm^−1^, 560 cm^−1.^ The results obtained by the FTIR spectroscopy TS1, TS2, CS1 and CS2 can be explained by the bonding property of carboxyl and hydroxide functional groups (Figures 10 and 11).

In Figure 8a, the small peak at 3665 cm^−1^ corresponds to the O‐H stretching for pure hydrogel PAM. The broader peaks at 1580.5 cm^−1^ and 1296 cm^−1^ correspond to stretching of O‐H for AgNP loaded hydrogel. Furthermore, the smaller peaks obtained at 497.6 cm^−1^ correspond to a small amount of (NO_3_) ^‐^ nitrate compounds. In Figure 8b, the lyophilised size reduced AgNP turmeric loaded hydrogel (TS1) shows an absorption band at 1321 cm^−1^ corresponding to the stretching frequency of carboxyl (COO)^‐^ groups. Similarly, the broad absorption band at 3194.1 cm^−1^ corresponds to stretching frequency of the O‐H groups. The band at 867.9 cm^−1^ occurs due to stretching vibrations of CH bonds. The band around the 867.97 cm^−1^ is obtained due to CH‐O‐CH_2_ bonds. Similarly, the bands around 675.09 cm^−1^ and 467.6 cm^−1^ show bending vibrations in the O‐H bonds and scissoring in the CH_2_ bonds. In Figure 8c, it is observed that ball milled size reduced AgNP‐turmeric loaded hydrogel (TS2) shows an absorption band at 1650 cm^−1^ corresponding to the stretching frequency of carboxyl (COO)^‐^ groups. The broad absorption band at 3320 cm^−1^ corresponds to the stretching frequency of the O‐H groups. The band at 1400 cm^−1^ occurs due to stretching vibrations of CH bonds. The band around the 950 cm^−1^ is obtained due to CH‐O‐CH_2_ bonds. Similarly, the bands around 780 cm^−1^ show bending vibrations in the O‐H bonds. From Figure 8, it was observed that the size reduced CS1 and CS2 have similar C‐H bonds as that of TS1 and TS2. However, there is difference in the FTIR bands for the samples. Figure 8d shows the broad absorption band at 3300 cm^−1^ corresponding to the stretching frequency of the O‐H groups. The bands around 1600 cm^−1^ and 1450 cm^−1^ occur due to stretching vibrations of CH bonds. In 3.2e, the band at 3720 cm^−1^ corresponds to the stretching frequency of carboxyl (COO) ^‐^ groups. The band around 2200 cm^−1^ shows scissoring in CH_2_ bonds. The band around 1285 cm ^−1^ corresponds to stretching frequency of the O‐H groups. The band at 560 cm^−1^ shows bending vibrations in O‐H bonds.

The Table 2 shows Fourier transform of certain composition of hydrogel samples. The first one with a composition of 45 mg of NaBH_4_, reducing agent and 5 mM of AgNP loaded hydrogel. The second one with a composition of 5 mg of turmeric and AgNP loaded hydrogel.

**TABLE 2:** FTIR values for TS1, TS2, CS1 and CS2

Sample	FTIR bands (cm)^−1^
**Ag nanoparticles loaded hydrogel**	3665, 1580.5, 1296, 497.6
**TS1**	3194.12, 1622.13, 1321.24, 867.97, 675.09, 467.6
**TS2**	3320, 1650, 1400, 859, 780
**CS1**	3300 cm^−1^, 1600 cm^−1^, 1450 cm^−1^
**CS2**	3720 cm^−1^, 2200 cm^−1^, 1285 cm^−1^, 560 cm^−1^

Abbreviation: FTIR, Fourier transform infrared spectroscopy.

Anti‐bacterial activity of TS1, TS2, CS1 and CS2 were tested on E‐coli and S. aureus bacteria and compared with S1 and S2. The analysis showed restrictions in formation of bacterial colonies in TS1, TS2, CS1 and CS2 compared with that of S1 and S2. From the results, it can be stated that the anti‐bacterial effects on the hydrogel are in the following order: TS1 and TS2 > CS1 and CS2 > Ag loaded hydrogels > S1 and S2.

**FIGURE 12:**
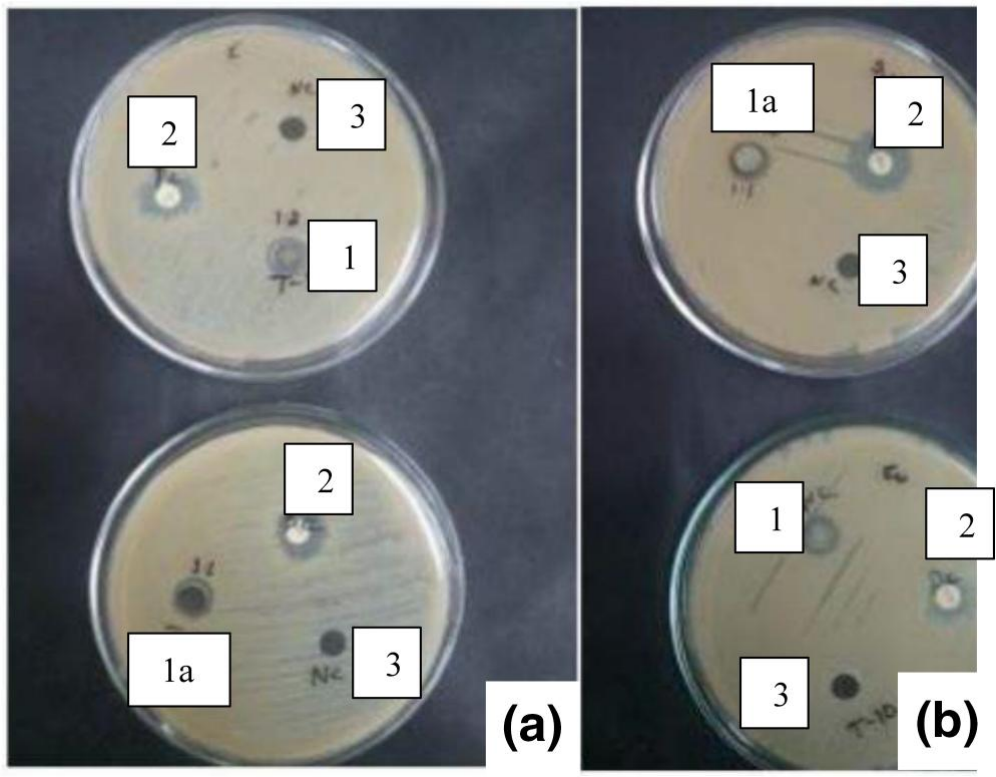
Results shown by TS1 and TS2 on (a) E‐coli bacteria and (b) Staphylococcus aureus bacteria

**FIGURE 13:**
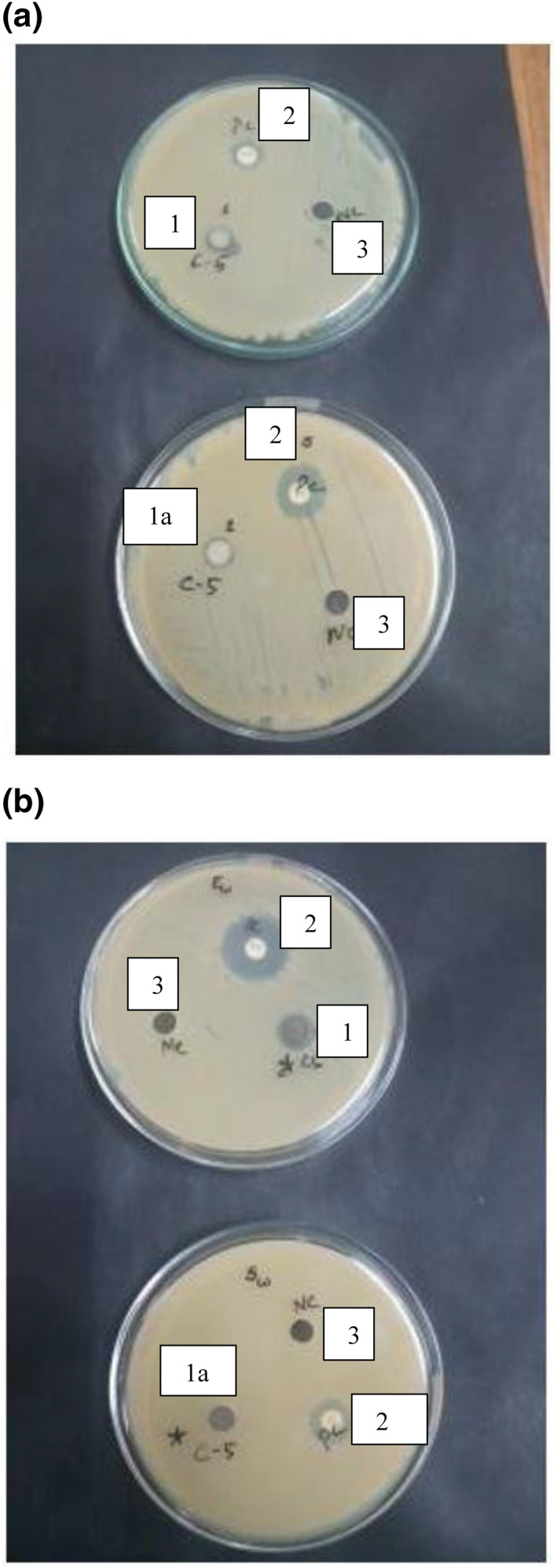
Results shown by CS1 and CS2 on (a) E‐coli bacteria and (b) Staphylococcus aureus bacteria

Figure 12 shows the zonal formation on MHA plates for turmeric‐Ag loaded hydrogels (TS1 and TS2) with water as the negative control. Table 3 represents the zonal formation results of TS1 and TS2 with water as the negative control on E‐coli bacteria and Staphylococcus aureus. Figure 13 represents the zonal formation on MHA plates for CS1 and CS2 with water as the negative control. Table 4 represents the zonal formation results of CS1 and CS2 with water as the negative control on E‐coli and Staphylococcus aureus (SA) bacteria.

**TABLE 3:** Zonal formation of TS1 and TS2 on E‐coli and S. Aureus

Name of the sample	Diameter of zonal (cm)‐ (E‐coli)		Diameter of zonal (cm) – S. Aureus	
	TS1	TS2	TS1	TS2
Test sample	1.2	1.1	1.1	No zone
Positive control (antibiotic)	1.5	1.5	1.5	1.5
Negative control(water)	No zone	No zone	No zone	No zone

**TABLE 4:** Zonal formation of CS1 and CS2 on E‐coli and S.aureus

Name of the sample	Diameter of zonal (cm) ‐ (E‐Coli)		Diameter of zonal (cm) – S. Aureus	
	CS1	CS2	CS1	CS2
Test sample	1	0.2	1	No zone
Positive control (antibiotic)	1.5	1.5	1.5	1.5
Negative control(water)	No zone	No zone	No zone	No zone

Samples in the Figure 9 tested for anti‐bacterial analysis : (1) TS1, (1a) TS2, (2) positive control or antibiotic and (3) negative control or water. The results obtained from the Table 3 show a zonal of 1.2  and 1.1 cm in TS1 and TS2 on E‐coli and a zonal of 1 cm and no zone in TS1 and TS2 for S. aureus bacteria.

Samples used in Figure 10 for the anti‐bacterial test analysis: 1) CS1, 1a) Reduced by CS2, 2) positive control or antibiotic and 3) negative control or water. Table 4 shows a zonal formation of 1  and 0.2 cm in CS1 and CS2 on E‐coli and a zonal formation of 1 cm and no zone in CS1 and CS2 for the S. aureus bacteria. This clearly explains that the bacterial resistance of TS1 and TS2 for E‐coli and Staphylococcus aureus bacteria is higher compared with CS1 and CS2.

## 5 CONCLUSION

Analysis of the size reduction of the Ag loaded hydrogel was carried out by lyophilisation and ball milling (S1 and S2) and comparison on the swelling behaviour of Ag loaded hydrogel, S1 and S2 was carried out at standard pH values. The swelling is higher in the case of Ag loaded hydrogel and S1 compared with S2. Above pH 7, the swelling ratio is similar for Ag loaded hydrogel and S1. SEM, UV‐Vis spectroscopy, XRD and FTIR analysis were carried out to characterise all the samples under study. Anti‐bacterial studies for E‐coli and S.aureus were carried on Ag loaded hydrogel, S1 and S2. The Ag loaded hydrogel performs better compared with that of S1 and S2. Furthermore, curcumin and turmeric with size reduced silver nanoparticle loaded hydrogel (TS1, TS2, CS1 and CS2) were tested for anti‐bacterial analysis. It was observed that in all the cases lyophilised hydrogels are more efficient than ball milled hydrogels and the effect of turmeric in TS1 and TS2 has better anti‐bacterial activity compared with curcumin, CS1 and CS2 for both E.Coli and S.aureus. Overall, TS1 showed better anti‐bacterial results stating they are strongly anti‐bacterial in nature and thus can be put to further use as a strong antibiotic in managing chronic wounds, by embedding them in bandages.
